# Prognostic Significance of Sarcopenia in Advanced Biliary Tract Cancer Patients

**DOI:** 10.3389/fonc.2020.01581

**Published:** 2020-09-02

**Authors:** Byung min Lee, Yeona Cho, Jun Won Kim, Hei Cheul Jeung, Ik Jae Lee

**Affiliations:** ^1^Department of Radiation Oncology, Yonsei Cancer Center, Yonsei University College of Medicine, Seoul, South Korea; ^2^Department of Radiation Oncology, Gangnam Severance Hospital, Yonsei University College of Medicine, Seoul, South Korea; ^3^Division of Medical Oncology, Gangnam Severance Hospital, Yonsei University College of Medicine, Seoul, South Korea

**Keywords:** biliary tract cancer, sarcopenia, inflammation, survival, prognosis

## Abstract

**Background:** Sarcopenia, systemic inflammation, and low muscularity significantly impact the survival of cancer patients. However, few studies have investigated how sarcopenia and systemic inflammation affect the prognosis of biliary tract cancer with distant metastasis. In this study, we investigated the association between sarcopenia with systemic inflammation and prognosis of metastatic biliary tract cancer.

**Materials and Methods:** Data collected from 353 metastatic biliary tract cancer patients from 2007 to 2016 were analyzed. To evaluate the skeletal muscle mass, computed tomography images at the upper level of the third lumbar vertebra (L3) were used. Sarcopenia was defined using the Japan Society of Hepatology guideline; L3 muscle index <42 cm^2^/m^2^ for male and <38 cm^2^/m^2^ for female patients. Systemic inflammation was evaluated using the neutrophil lymphocyte ratio (NLR). Patients with NLR > 3 were categorized into the inflammatory category. The overall survival (OS) and progression free survival (PFS) were analyzed. Subgroup analysis was performed on those who received gemcitabine/cisplatin (GP) chemotherapy and depending on the presence of sarcopenia and inflammation.

**Results:** Patients with sarcopenia showed lesser 1-year OS than those without (25.5 vs. 38.2%, *p* = 0.019). The patients with high NLR (NLR > 3) were associated with a shorter OS than were those with a low NLR (NLR ≤ 3) (21.0 vs. 52.8%, *p* < 0.001). Based on these results, we categorized the patients into three groups; sarcopenia accompanied by high NLR, no sarcopenia and low NLR, and either sarcopenia or high NLR. The OS of patients was well-stratified according to this grouping (1-year OS; 18.3 vs. 30.3 vs. 55.8%, *p* < 0.001). Concordant with OS results, the PFS was well-stratified based on the presence of either sarcopenia or high NLR (Sarcopenia; 9.5 vs. 19.4%, *p* < 0.001, NLR; 10.0 vs. 23.4%, *p* < 0.001). The PFS was significantly associated with high NLR and sarcopenia (1-year PFS; 7.8 vs. 13.0 vs. 27.9%, *p* < 0.001).

**Conclusion:** Sarcopenia with inflammation was associated with inferior OS and PFS. In addition, sarcopenia accompanied by inflammation was associated with poor prognosis. Conservative treatments such as nutritional support, exercise, and pharmacologic intervention could help metastatic biliary tract cancer patients to overcome sarcopenia and the inflammatory status.

## Introduction

Sarcopenia, the loss of skeletal mass and strength, is part of the normal aging process as well as other health problems such as liver cirrhosis, renal failure, cognitive problems, and cancer (1, 2). The importance of sarcopenia in cancer has been increasingly recognized, as low muscularity is a significant predictor of poor prognosis in various cancers (3–5).

Studies have shown that low skeletal muscle mass before surgery was significantly associated with overall survival (OS) in biliary tract cancer (BTC) patients (6, 7). Only few reports have reported sarcopenia as a prognostic factor for advanced BTC. If loss of skeletal muscle mass occurs, the tolerance to anticancer treatment is reduced, which is associated with reduced survival (8, 9). However, the mechanism of sarcopenia in malignancy is not fully defined (10). Available literature suggests that sarcopenia in patients with malignancy is related to inflammation as well as older age and poor performance (9, 11). Low muscularity of patients could lead to inflammation around the muscle and can contribute to systemic inflammation (12).

Due to limited knowledge on the mechanism of sarcopenia, the clinical management of sarcopenia is limited and complex (10, 13, 14). Over the past few decades, our understanding of sarcopenia has improved, but there is still a lack of a definition and diagnostic criteria for sarcopenia.

Several studies have demonstrated that systemic inflammation is related to poor prognosis (15, 16). To evaluate the systemic inflammatory status, common inflammatory markers such as neutrophil/lymphocyte ratio (NLR), platelet/lymphocyte ratio (PLR), and C-reactive protein (CRP) were used. Previous studies showed that colorectal, small cell lung, and head and neck cancer patients with high NLR and low skeletal muscle mass have an inferior OS (17–19). In this study, we investigated whether sarcopenia accompanied by systemic inflammation affected the overall survival in advanced BTC patients.

## Patients and Methods

### Patient Population

We retrospectively reviewed the data of advanced BTC patients from a single institution. Patients with gallbladder cancer, intrahepatic, perihilar, extrahepatic bile duct cancer, and ampulla of Vater cancer were included in this study, and those with distant metastasis at initial diagnosis were analyzed.

Totally 353 patients diagnosed metastatic BTC in Gangnam Severance Hospital from January 2007 to November 2016 met the inclusion criteria. The diagnosis was made through tissue biopsy or cytology. The inclusion criteria for this study were as follows: (1) age over 18 years; (2) diagnosis of BTC via histologic confirmation; (3) metastatic BTC at diagnosis; and (4) patients with available medical records.

The exclusion criteria were as follows: (1) patients with widespread brain or leptomeningeal metastasis; (2) uncontrolled infections or poor medical conditions; (3) synchronous malignancies; (4) patients lost at follow-up; and (5) patients where the tissue area at the third lumbar level could not be measured or patients without height data. This study was approved by the institutional review board of the Gangnam Severance Hospital (3-2019-0257). Informed consent was not required owing to the retrospective nature of the study.

### Measurement of Body Composition and Definition of Sarcopenia

The previously validated computed tomography (CT)-based body composition measurement method was used to identify if a patient had sarcopenia. We selected a single axial slice at the upper border of third lumbar spine vertebra (L3) level for measurement. The delineation of skeletal muscle, visceral fat, and subcutaneous fat tissue was performed using the MIM Vista software (MIM corp., Version 6.6.14, OH, USA) based on Hounsfield units (HUs). The threshold of HUs was applied as follows: Skeletal muscle (−29 to +150 HU); visceral fat tissue (−150 to −50 HU); and subcutaneous fat tissue (−190 to −30 HU). The measurements for sarcopenia were performed by a single radiation oncologist (B. M. Lee). All other researchers involved in this study were blinded to the outcome of measurements.

**Figure 1:**
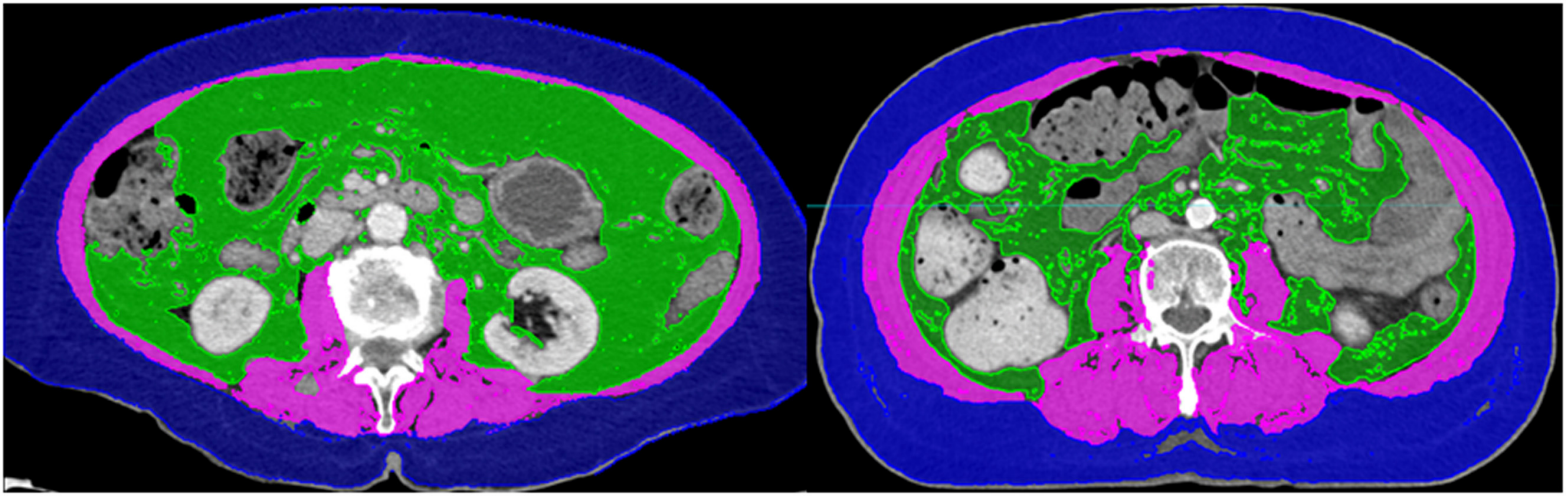
Computed tomography images of patients with (Left) and without sarcopenia (Right). The two patients had similar body mass indices.

To determine the amount of skeletal muscle, the L3 skeletal muscle index was used. First, the cross-sectional volume at the L3 level was divided by the thickness of the axial slice to get the cross-sectional area. The cross-sectional areas were divided by the height of the patients to obtain the L3 skeletal muscle index. According to international consensus, sarcopenia is defined as an L3 muscle index of <55 and 39 cm^2^/m^2^ for male and female patients, respectively (20). However, the studies contributing to this consensus were mostly based on the European and American guidelines (21, 22). As the patients included in this study were of Asian descent, the Japan Society of Hepatology (JSH) guideline was used and sarcopenia was defined as L3 muscle index <42 cm^2^/m^2^ for male and 38 cm^2^/m^2^ for female patients (23). Figure 1 demonstrates the CT images of patients with sarcopenia and without sarcopenia.

### Indicator of Inflammatory Status

The NLR was used to evaluate the inflammatory status of patients, and calculated by dividing the neutrophil count by the lymphocyte count. Every patients underwent the complete blood count before the administering the chemotherapy and based on this examination results, we calculated the NLR value. The optimal cut off values for NLR were different in each study (24, 25). We adopted the cut off value of NLR that was previously used for metastatic BTC (25).

### Statistical Analysis

The Fisher's exact test or χ^2^-test was used to analyze categorical data. For continuous data, the Mann–Whitney *U*-test was used for comparison. OS was defined as the time from the date of diagnosis to either death due to any cause or to last follow-up. The progression free survival (PFS) was defined as the time from the date of diagnosis to date of either disease progression or death. The survival curves were evaluated using the Kaplan–Meier method. The univariate and multivariate analyses were performed using the Cox proportional hazards model to determine the association between OS, PFS, and the factors we suggested. The multivariate analysis was conducted using the variables that were significant predictors of OS and PFS in the univariate analysis with backward stepwise. The hazard ratios (HRs) and 95% confidence intervals (CIs) were calculated. A *p* < 0.05 was considered statistically significant. The analyses were conducted using IBM SPSS version 25.0 (SPSS, Chicago, IL, USA).

## Results

### Patient Characteristics

A total of 353 patients were included with a median follow-up of 7.77 months [interquartile range (IQR): 3.27–14.70]. Table 1 shows the overall patients' characteristics. The median age was 67 years (IQR: 58–75), and there were 203 male patients (57.5%). Of the 353 patients, 158 (44.9%) showed good performance status with ECOG (Eastern Cooperative Oncology Group) 0 or 1, while 194 (55.1%) had poor performance status (ECOG ≥2). Histologically, 202 tumors (74.8%) were either well-differentiated or moderately differentiated.

**Table 1:** Patient characteristics.

**Variables**	**No**.	**%**
Age (median, IQR)	67 (58–75)	
<67	166	47.00%
≥67	187	53.00%
**Sex**		
Male	203	57.50%
Female	150	42.50%
**Primary**		
Gall bladder	130	36.80%
Intrahepatic bile duct	112	31.70%
Non-hilar bile duct	43	12.20%
Perihilar bile duct	58	16.40%
Ampullary	10	2.90%
**Pathology**		
WD/MD	202	74.80%
PD	68	25.20%
**Performance status**		
ECOG 0	27	7.70%
ECOG 1	131	37.20%
ECOG 2	141	40.10%
ECOG 3	37	10.50%
ECOG 4	16	4.50%
CA 19-9 (median, IQR)	311.85 (36.65–3262.2)
CA 19-9 normal	85	24.10%
CA 19-9 elevated	267	75.90%
CEA (median, IQR)	4.45 (2.20–24.80)
CEA normal	187	53.10%
CEA elevated	165	46.90%
**Albumin**		
≥3.4 (g/dL)	259	73.40%
<3.4 (g/dL)	94	26.60%
**Protein**		
≥6.9 (g/dL)	186	52.70%
<6.9 (g/dL)	167	47.30%
**Cholesterol**		
<139 (mg/dL)	91	25.80%
≥139 (mg/dL)	262	74.20%
**BUN**		
<23.0 (mg/dL)	304	86.10%
≥23.0 (mg/dL)	49	13.90%
**Bilirubin**		
<1.2 (mg/dL)	192	54.50%
≥1.2 (mg/dL)	160	45.50%
**C-reactive protein**		
<8.0 (mg/L)	100	29.00%
≥8.0 (mg/L)	245	71.00%
**Neutrophil lymphocyte ratio**		
≤3.0	129	36.80%
>3.0	222	63.20%

*IQR, interquartile range; WD, well-differentiated; MD, moderately differentiated; PD, poorly differentiated; ECOG, Eastern Cooperative Oncology Group; CA 19-9, carbohydrate antigen 19-9; CEA, carcinoembryonic antigen; BUN, blood urea nitrogen*.

We divided the patients into two groups according to the presence of sarcopenia. Table 2 compares the characteristics between the patients with and without sarcopenia. Sarcopenia was associated with older age (71 vs. 65-years, *p* < 0.001) and female sex (51.6 vs. 35.1%, *p* = 0.002). The sarcopenia group had more patients with poor performance status (ECOG ≥2) than the non-sarcopenia group (68.4 vs. 44.3%, *p* < 0.001). There were significant differences in the blood chemistry profile between the two groups. There were more patients with hypoalbuminemia and hypoproteinemia in the sarcopenia group (hypoalbuminemia: 34.6 vs. 20.1%, *p* = 0.002; hypoproteinemia: 53.5 vs. 42.3%, *p* = 0.036). There were more patients with NLR > 3 in the sarcopenia group (72.8 vs. 55.4%, *p* < 0.001).

**Table 2:** Comparison of patient characteristics.

**Variables**	**Sarcopenia (*****n*** **=** **159)**		**Non-sarcopenia (*****n*** **=** **194)**		***p*-value**
	**No**.	**%**	**No**.	**%**	
Age (median, IQR)	71 (62–79)		65 (56–71)		
<67	53	33.30%	113	58.20%	<0.001
≥67	106	66.70%	81	41.80%	
**Sex**					
Male	77	48.40%	126	64.90%	0.002
Female	82	51.60%	68	35.10%	
**Primary**					
Gall bladder	56	35.20%	74	38.10%	0.618
Intrahepatic bile duct	53	33.30%	59	30.40%	
Non-hilar bile duct	16	10.10%	27	13.90%	
Perihilar bile duct	28	17.60%	30	15.50%	
Ampullary	6	3.80%	4	2.10%	
**Pathology**					
WD/MD	79	69.90%	123	78.30%	0.115
PD	34	30.10%	34	21.70%	
**Performance status**					
ECOG 0	7	4.40%	20	10.30%	<0.001
ECOG 1	43	27.20%	88	45.40%	
ECOG 2	75	47.50%	66	34.00%	
ECOG 3	22	13.90%	15	7.70%	
ECOG 4	11	7.00%	5	2.60%	
CA 19-9 (median, IQR)	373.9 (44.9–3190.0)		241.1 (29.9–3494.7)		
CA 19-9 normal	34	21.40%	51	26.40%	0.271
CA 19-9 elevated	125	78.60%	142	73.60%	
CEA (median, IQR)	7.30 (2.60–25.00)		3.70 (1.90–24.50)		
CEA normal	75	47.20%	112	58.00%	0.042
CEA elevated	84	52.80%	81	42.00%	
**Albumin**					
≥3.4 (g/dL)	104	65.40%	155	79.90%	0.002
<3.4 (g/dL)	55	34.60%	39	20.10%	
**Protein**					
≥6.9 (g/dL)	74	46.50%	112	57.70%	0.036
<6.9 (g/dL)	85	53.50%	82	42.30%	
**Cholesterol**					
<139 (mg/dL)	42	26.40%	49	25.30%	0.805
≥139 (mg/dL)	117	73.60%	145	74.70%	
**BUN**					
<23.0 (mg/dL)	134	84.30%	170	87.60%	0.365
≥23.0 (mg/dL)	25	15.70%	24	12.40%	
**Bilirubin**					
<1.2 (mg/dL)	85	53.50%	107	55.40%	0.710
≥1.2 (mg/dL)	74	46.50%	86	44.60%	
**C-reactive protein**					
<8.0 (mg/L)	43	27.40%	57	30.30%	0.550
≥8.0 (mg/L)	114	72.60%	131	69.70%	
**Neutrophil lymphocyte ratio**					
≤3.0	43	27.20%	86	44.60%	0.001
>3.0	115	72.80%	107	55.40%	

*IQR, interquartile range; WD, well-differentiated; MD, moderately differentiated; PD, poorly differentiated; ECOG, Eastern Cooperative Oncology Group; CA 19-9, carbohydrate antigen 19-9; CEA, carcinoembryonic antigen; BUN, blood urea nitrogen*.

### Analysis of Overall Survival and Prognostic Factors

**Figure 2:**
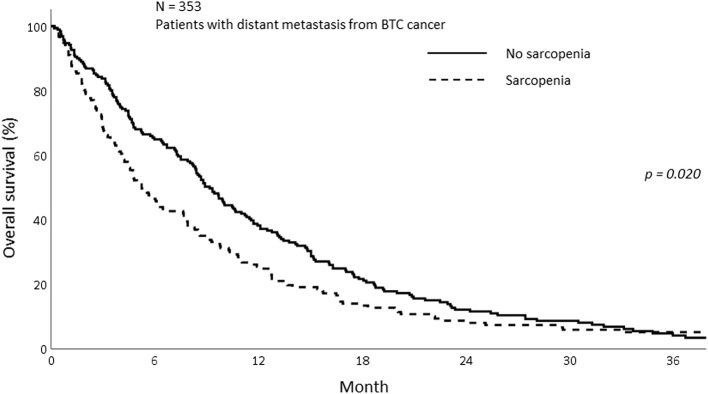
Overall survival of patients with and without sarcopenia.

**Figure 3:**
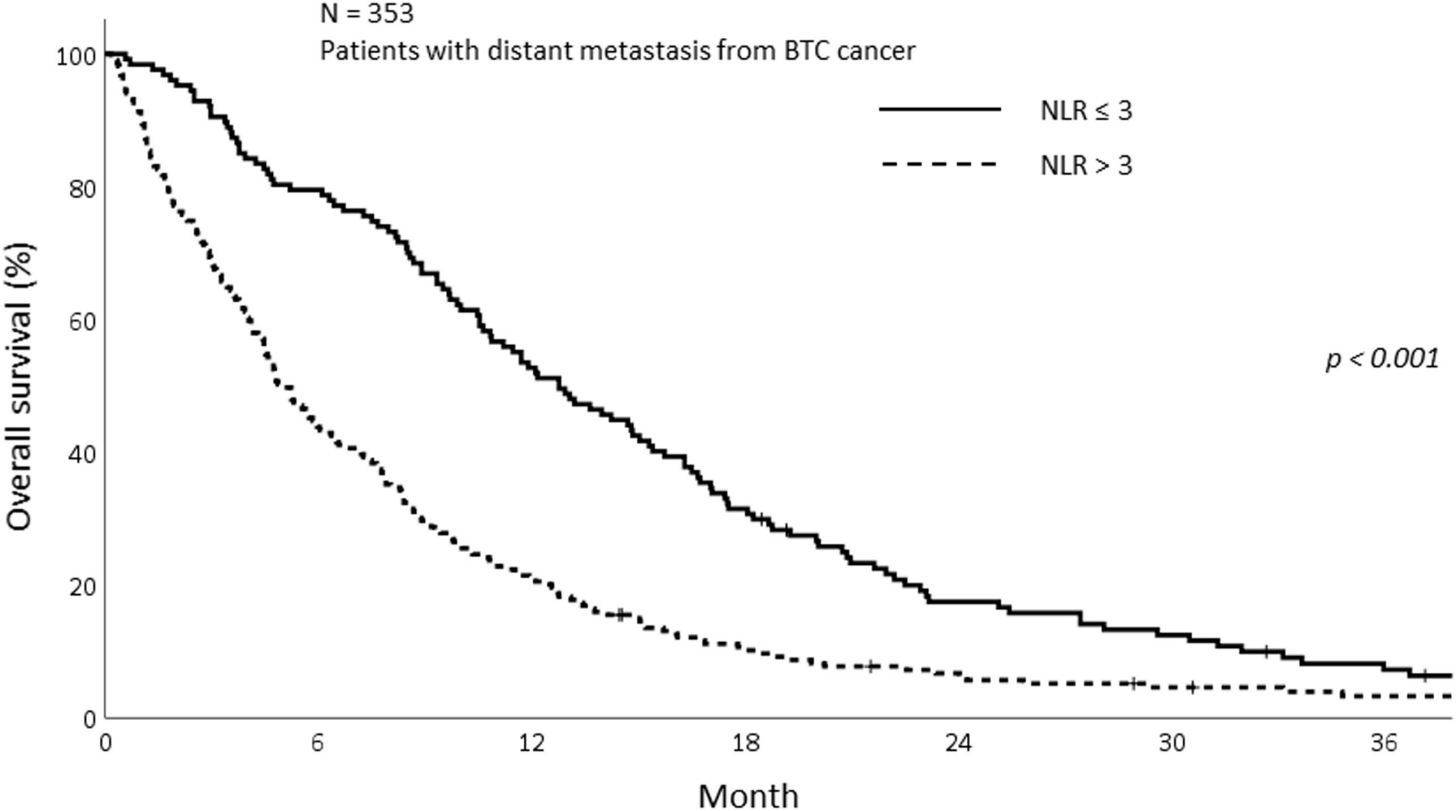
Overall survival of patients with high and low NLR.

The median OS of all patients in this study was 7.77 months (IQR; 3.27–14.70). The median OS was 5.23 and 8.90 months in the sarcopenia and non-sarcopenia groups, respectively (*p* = 0.057). The 1-year OS was significantly different between those with and without sarcopenia (25.5 vs. 38.2%, *p* = 0.020; Figure 2). As the mechanism of sarcopenia in cancer patients was known to be associated to cancer-related inflammation, we assessed the effect of systemic inflammation on survival using NLR. The 1-year OS for patients with NLR > 3 was 21.0% whereas for those with NLR ≤ 3 was 52.8% (*p* < 0.001; Figure 3).

**Figure 4:**
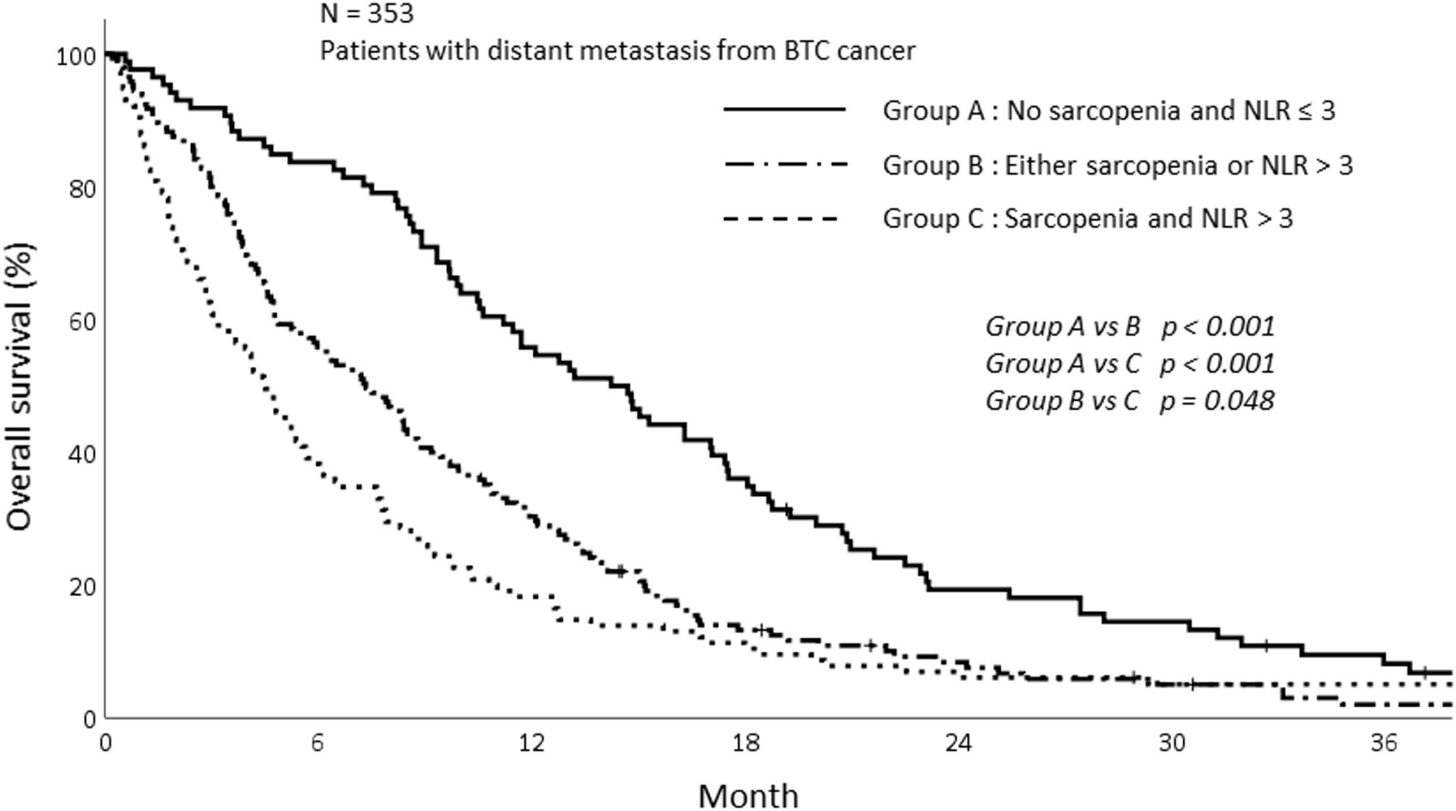
Overall survival of patients depending on sarcopenia and NLR status.

Based on this result, we analyzed survival depending on sarcopenia and inflammatory status. The patients were stratified into three groups according to sarcopenia and NLR as follows: patients with no sarcopenia and NLR ≤ 3, patients with sarcopenia and NLR > 3, and patients with either sarcopenia or NLR > 3. The survival of patients with sarcopenia and NLR > 3 was significantly poorer than those without sarcopenia and NLR ≤ 3. The 1-year OS for patients showing NLR ≤ 3 and without sarcopenia was 55.8%, while the 1-year OS for the group with NLR > 3 and sarcopenia and either sarcopenia or NLR > 3 was 18.3 and 30.3%, respectively (*p* < 0.001; Figure 4).

The results of univariate and multivariate analysis are summarized in Table 3. In univariate analysis, 12 variables were factors associated with survival. The 12 variables were as follows; sex, pathology, ECOG, carbohydrate antigen 19-9 (CA 19-9), CRP, albumin, protein, cholesterol, BUN, NLR, Sarcopenia, group stratification based on sarcopenia and NLR. Among these 12 variables, we excluded the NLR and sarcopenia since there were significant correlation between group stratification and both sarcopenia status and NLR. Totally, 10 independent variables were included in the multivariate logistic regression analysis using backward stepwise (17). In the multivariate analysis, group with sarcopenia and high NLR (*p* = 0.004) were significantly associated with poor OS along with male patients (*p* = 0.010), patients with higher CA 19-9 (*p* = 0.032), and those with poor ECOG status (*p* < 0.001).

**Table 3:** Univariate and multivariate analysis of overall survival.

**Variables**	**Univariate analysis**			**Multivariate analysis**		
	**HR**	**95% CI**	***p*-value**	**HR**	**95% CI**	***p*-value**
Sex (male vs. female)	1.24	1.00–1.55	0.050	1.41	1.09–1.84	0.010
Age (<67 vs. ≥67)	1.16	0.93–1.44	0.185			
Pathology (WD/MD vs. PD)	1.53	1.15–2.03	0.003			
ECOG			<0.001			<0.001
ECOG 0 vs. ECOG 1	2.12	1.32–3.40	0.002	1.99	1.17–3.38	0.011
ECOG 0 vs. ECOG 2	2.98	1.85–4.78	<0.001	2.52	1.47–4.32	0.001
ECOG 0 vs. ECOG 3	6.84	3.94–11.88	<0.001	6.17	3.16–12.02	<0.001
ECOG 0 vs. ECOG 4	14.28	7.28–28.02	<0.001	5.98	1.92–18.61	0.002
CA 19-9 (per 100)	1.00	1.00–1.01	<0.001	1.00	1.00–1.004	0.032
CEA (per 20)	1.00	1.00–1.01	0.066			
CRP (normal vs. elevated)	1.86	1.45–2.39	<0.001			
Albumin (≥3.4 vs. <3.4)	1.97	1.54–2.51	<0.001			
Protein (≥6.9 vs. <6.9)	1.43	1.15–1.77	0.001			
Cholesterol (<139 vs. ≥139)	0.66	0.52–0.85	0.001			
BUN (<23.0 vs. ≥23.0)	1.45	1.07–1.97	0.018			
Bilirubin (<1.2 vs. ≥1.2)	0.99	0.80–1.23	0.920			
NLR (<3.00 vs. ≥3.00)	1.94	1.55–2.43	<0.001			
Sarcopenia (Yes vs. No)	0.77	0.62–0.96	0.020			
VATI (low vs. high)	0.98	0.79–1.22	0.860			
SATI (low vs. high)	0.86	0.69–1.06	0.156			
BMI (<25 vs. ≥25)	0.81	0.63–1.03	0.085			
Sarcopenia and NLR			<0.001			0.004
Low NLR and no sarcopenia vs. either	1.69	1.28–2.22	<0.001	1.60	1.16–2.22	0.005
Low NLR and no sarcopenia vs. high NLR and sarcopenia	2.13	1.59–2.84	<0.001	1.80	1.25–2.59	0.002

*HR, hazard ratio; CI, confidence interval; CBD, common bile duct; CCC, cholangiocarcinoma; GB, gallbladder; WD, well-differentiated; MD, moderately differentiated; PD, poorly differentiated; ECOG, Eastern Cooperative Oncology Group; CA 19-9, carbohydrate antigen 19-9; CEA, carcinoembryonic antigen; CRP, C-reactive protein; BUN, blood urea nitrogen; NLR, neutrophil lymphocyte ratio; VATI, visceral adipose tissue index; SATI, subcutaneous adipose tissue index*.

### Progression Free Survival and Prognostic Factors

**Figure 5:**
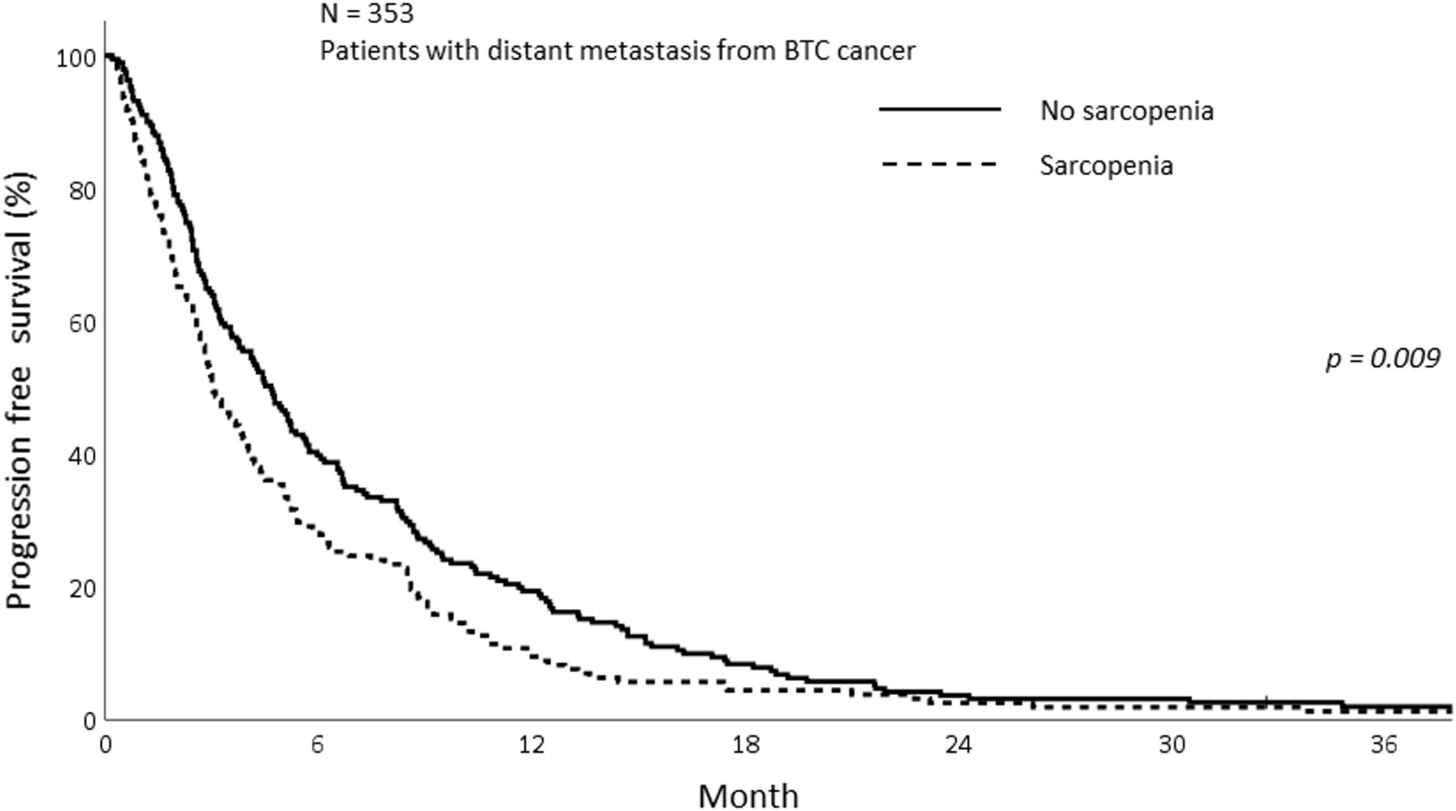
Progression free survival of patients with and without sarcopenia.

**Figure 6:**
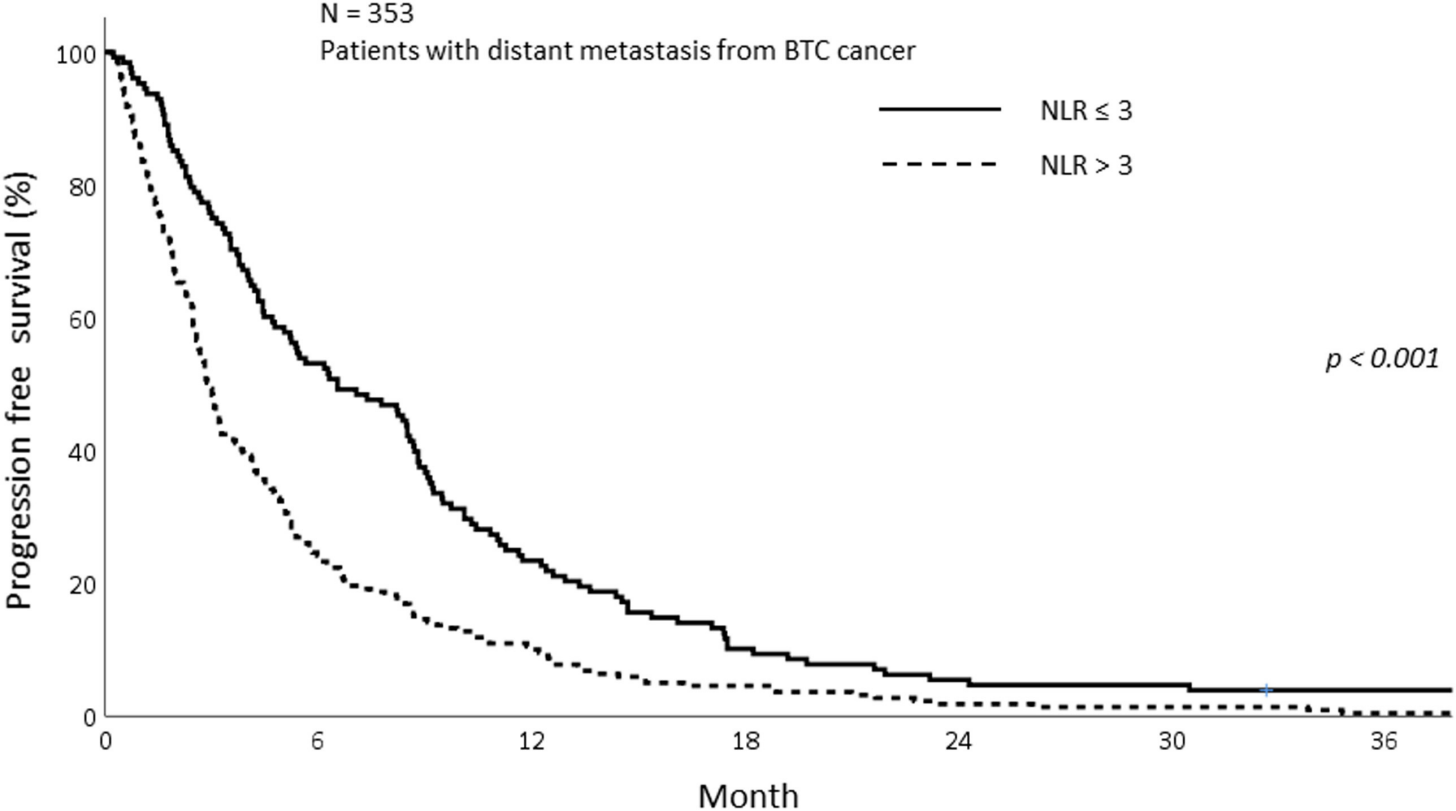
Progression free survival of patients with high and low NLR.

**Figure 7:**
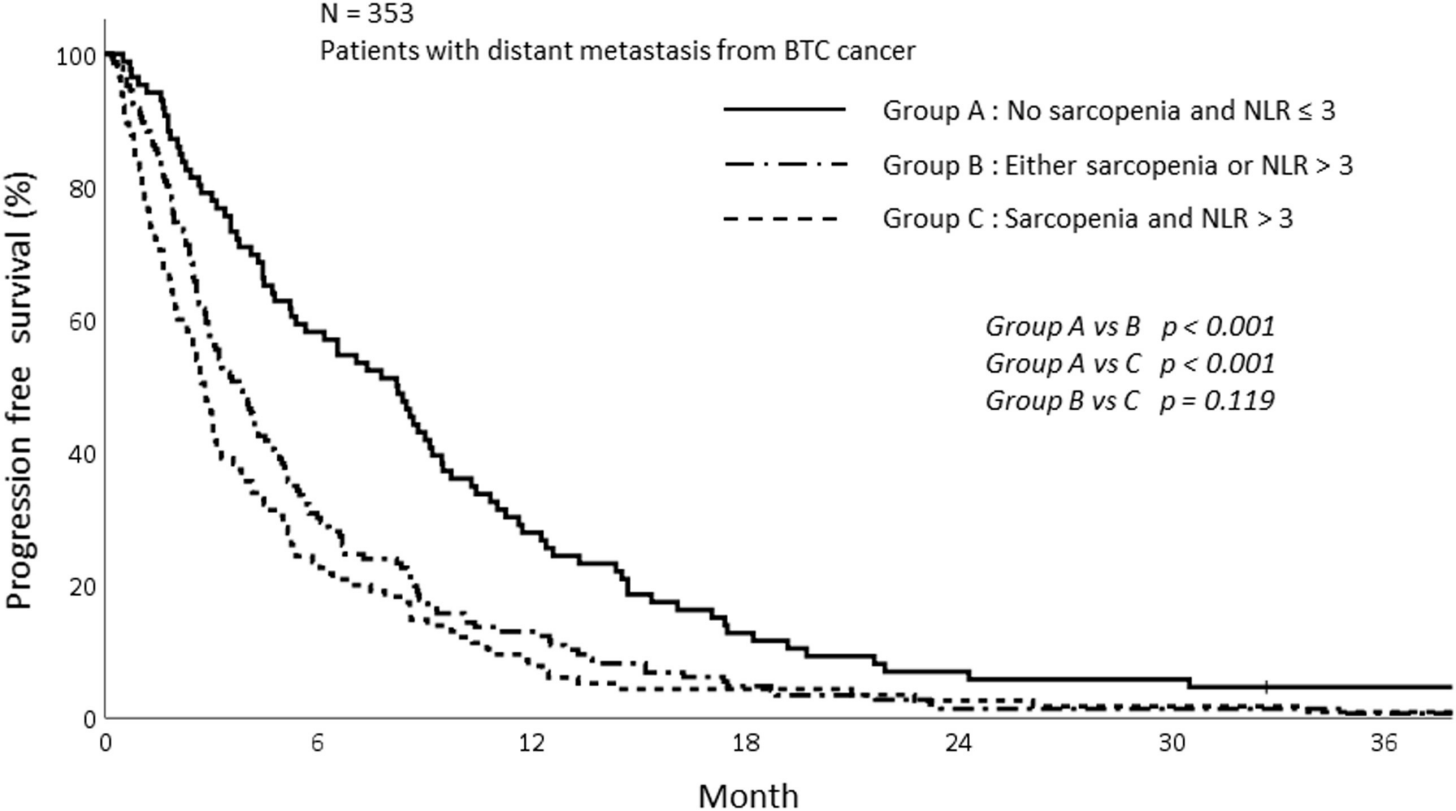
Progression free survival of patients depending on sarcopenia and NLR status.

The PFS was analyzed according to sarcopenia and inflammatory status. As shown in Figure 5, sarcopenia was associated with inferior PFS (1-year PFS; 9.5 vs. 19.4%, *p* < 0.009). In addition, patients with NLR > 3 showed inferior PFS compared to those with NLR ≤ 3 (1-year PFS; 10.0 vs. 23.4%, *p* < 0.001; Figure 6). Patients with sarcopenia and high NLR demonstrated lesser PFS than the other two groups (1-year PFS; 7.8 vs. 13.0 vs. 27.9%, *p* < 0.001; Figure 7).

In the univariate analysis, NLR, sarcopenia, and group depending on NLR status and sarcopenia were significantly associated with PFS. Sarcopenia with NLR > 3 was a significant predictor of poor PFS. Along with high NLR and sarcopenia, poorly differentiated carcinoma (*p* = 0.017), poor performance (*p* < 0.001), and high CA 19-9 (*p* = 0.011) were significant predictors of poor PFS (Table 4).

**Table 4:** Univariate and multivariate analysis of progression free survival.

**Variables**	**Univariate analysis**			**Multivariate analysis**		
	**HR**	**95% CI**	***p*-value**	**HR**	**95% CI**	***p*-value**
Sex (male vs. female)	1.24	1.00–1.542	0.047			
Age (<67 vs. ≥67)	1.04	0.84–1.29	0.717			
Pathology (WD/MD vs. PD)	1.67	1.26–2.21	<0.001	1.44	1.07–1.95	0.017
ECOG			<0.001			<0.001
ECOG 0 vs. ECOG 1	1.68	1.09–2.59	0.018	1.48	0.92–2.39	0.110
ECOG 0 vs. ECOG 2	2.01	1.31–3.08	0.001	1.60	0.98–2.60	0.058
ECOG 0 vs. ECOG 3	4.49	2.69–7.49	<0.001	3.94	2.08–7.47	<0.001
ECOG 0 vs. ECOG 4	6.23	3.30–11.77	<0.001	2.02	0.67–6.03	0.210
CA 19-9 (per 100)	1.00	1.00–1.00	0.001	1.00	1.00–1.01	0.011
CEA (per 20)	1.00	0.99–1.01	0.220			
CRP (normal vs. elevated)	1.71	1.34–2.18	<0.001			
Albumin (≥3.4 vs. <3.4)	1.59	1.25–2.02	<0.001			
Protein (≥6.9 vs. <6.9)	1.34	1.09–1.66	0.006			
Cholesterol (<139 vs. ≥139)	0.71	0.55–0.90	0.005			
BUN (<23.0 vs. ≥23.0)	1.23	0.90–1.67	0.191			
Bilirubin (<1.2 vs. ≥1.2)	0.89	0.72–1.11	0.302			
NLR (<3.00 vs. ≥3.00)	1.75	1.40–2.18	<0.001			
Sarcopenia (Yes vs. No)	0.75	0.61–0.93	0.009			
VATI (low vs. high)	0.92	0.74–1.13	0.416			
SATI (low vs. high)	0.88	0.71–1.09	0.246			
BMI (<25 vs. ≥25)	0.85	0.67–1.08	0.195			
Sarcopenia and NLR			<0.001			0.015
Low NLR and no sarcopenia vs. either	1.67	1.27–2.18	<0.001	1.49	1.09–2.04	0.014
Low NLR and no sarcopenia vs. high NLR and sarcopenia	2.00	1.50–2.65	<0.001	1.64	1.15–2.34	0.007

*HR, hazard ratio; CI, confidence interval; CBD, common bile duct; CCC, cholangiocarcinoma; GB, gallbladder; WD, well-differentiated; MD, moderately differentiated; PD, poorly differentiated; ECOG, Eastern Cooperative Oncology Group; CA 19-9, carbohydrate antigen 19-9; CEA, carcinoembryonic antigen; CRP, C-reactive protein; BUN, blood urea nitrogen; NLR, neutrophil lymphocyte ratio; VATI, visceral adipose tissue index; SATI, subcutaneous adipose tissue index*.

### The Subgroup Analysis of OS and PFS for Patients Who Received GP Chemotherapy

A subgroup analysis was performed for patients who had received gemcitabine/cisplatin (GP) based chemotherapy. Of the 353 patients, 132 received GP chemotherapy. The median follow-up period was 10.67 months (IQR; 5.97–18.48). The OS and PFS rates for patients who received GP chemotherapy were evaluated depending on sarcopenia and inflammatory status.

The OS and PFS were not significantly different between the sarcopenia and non-sarcopenia groups among patients who received GP chemotherapy. The 1-year OS for sarcopenia and non-sarcopenia patients were 42.6 and 50.7%, respectively (*p* = 0.844; Supplementary Figure 1). The 1-year PFS was 12.7 and 28.0% for sarcopenia and non-sarcopenia patients, respectively (*p* = 0.123; Supplementary Figure 2). The patients with NLR > 3 had significantly poorer OS and PFS than patients with NLR ≤ 3 (1-year OS; 63.0 vs. 36.5%, *p* = 0.003, 1-year PFS; 27.3 vs. 17.6%, *p* = 0.008; Supplementary Figures 3, 4).

The results of univariate and multivariate analysis in OS among patients who received GP chemotherapy are shown in Supplementary Table 1. The OS was not affected by the sarcopenia (*p* = 0.844) but was affected by the high NLR (*p* = 0.003) in the univariate analysis. The multivariate analysis also showed the relevance of OS and high NLR (*p* = 0.019). The results of univariate and multivariate analysis of PFS are shown in Supplementary Table 2. Sarcopenia was not associated with PFS but the inflammation status was significantly associated with PFS (*p* = 0.003). The NLR status remained significant for predicting PFS in multivariate analysis (*p* = 0.019).

### Analysis of Subcutaneous Adipose Tissue Index and Visceral Adipose Tissue Index

To evaluate the prognostic significance depending on subcutaneous adipose tissue index (SATI) and visceral adipose tissue index (VATI), we compared the OS according to high and low SATI and VATI. The cut off value for VATI and SATI were determined using median values. The VATI cut off values were 29.5 and 28.5 cm^2^/m^2^ for female and male patients, respectively. The cut off values of SATI were 56.5 and 26.5 cm^2^/m^2^ for female and male patients, respectively. More sarcopenia than non-sarcopenia patients had either low VATI or low SATI (VATI; 57.2 vs. 41.8%, *p* = 0.004, SATI; 61.0 vs. 36.6%, *p* < 0.001).

We compared the OS of patients depending on VATI and SATI. There was no difference in OS between patients with high VATI and low VATI (1-year OS: 34.6 vs. 30.2%, *p* = 0.860). In addition, concordant with VATI results, SATI was not a significant factor for OS. The 1-year OS for high and low SATI was 35.0 and 29.7% (*p* = 0.155), respectively. Altogether, the adipose index was not associated with OS in metastatic BTC.

## Discussion

In this study, we demonstrated that sarcopenia was associated with poor OS and PFS in BTC patients with distant metastasis at diagnosis. Furthermore, high NLR, which indicates inflammatory status, was also associated with reduced OS and PFS. Based on these two results, we stratified the patients into three groups. The patients with both sarcopenia and high NLR showed the poorest OS and PFS compared to those without sarcopenia and low NLR (NLR ≤ 3), and those with either sarcopenia or high NLR.

There is increasing evidence that the loss of muscle may affect the prognosis of cancer (4, 5). As patients with malignancy are generally more vulnerable to degenerative conditions, the decrease in muscle mass, and dysfunction could be easily identified. In particular, patients with malignancy that have progressed to the unresectable or metastatic stage are more likely to be affected by sarcopenia (26). The relevance between sarcopenia and poor prognosis had been shown in breast (5), lung (3), esophageal (27), hepatocellular (28), and colon cancers (29).

Yoon et al. used two ways to evaluate the sarcopenia status of BTC patients; skeletal muscle attenuation; and index. They also suggested that those with low skeletal muscle attenuation had negative influence on survival when compared to those that underwent resection for BTC (30).

In our study, sarcopenia alone did not worsen the PFS and OS in multivariate analysis. However, sarcopenia was significantly associated with poor prognosis in the univariate analysis. Concordant with our data, Yoon et al., who analyzed the significance of sarcopenia on BTC, showed that a low skeletal muscle index was not associated with improved survival in a multivariate analysis (30). It is possible that BTC is more affected by tumor specific factors rather than patient related factors such as sarcopenia.

It remains controversial as to what the cut off values of sarcopenia should be. Sarcopenia is related to several factors such as age, sex, ethnicity, and the region of the body used for measurements. We adopted the cut off value from the JSH guidelines which is based on Asian patients with liver disease (23). As the amount of muscle wasting is different depending on disease and ethnicity, further study would be helpful to clarify the cut off value of sarcopenia in metastatic BTC.

Systemic inflammation is one of the crucial parameters that can predict the cancer outcome in multiple cancers. Many inflammatory markers, such as CRP, NLR, and PLR have been associated with poor prognosis for various cancers (31–33).

Inflammation facilitates cancer progression through the activation of phosphatidylinositol 3-kinase and the recruitment of metalloproteinase-9, which promote cancer cell proliferation, inhibit cell apoptosis (34), and promote angiogenesis and tumor migration (35). Evidently, high NLR, which is associated with inflammation, is linked to poor prognosis and poor response to treatment, and has been demonstrated in various cancers including melanoma (36), colorectal cancer (37, 38), intrahepatic cholangiocarcinoma (39), prostate cancer (40), and pancreatic cancer (41).

In BTC, the NLR cut off value of 3 is frequently used to evaluate the inflammatory status. Several studies have compared NLR values with OS in BTC. In these studies, the patients with NLR > 3 had shorter OS than patients with NLR ≤ 3 (Median OS; 21.6 vs. 12.0 months, *p* = 0.01). Patients with advanced stage had more predictive NLR status than the surgical group (24, 25). In our study, the OS was significantly different depending on NLR status.

Notably, patients with inflammation accompanied by sarcopenia were associated with poor prognosis. These patients showed poor OS rates and more disease progression than those without inflammation and sarcopenia. The relationship between systemic inflammation and waste of muscle mass is gathering increased attention over the recent years (19). There is a close connection between inflammatory markers and the activation of catabolic pathway (42). For instance, the tumor necrosis factor (TNF) and interleukin 6 (IL-6), which are generated from the tumors and the surrounding cells, can hasten both protein degradation and also inhibit protein synthesis (43). Furthermore, the tumor itself promotes inflammation, which tends to facilitate tumor progression. The secretion of proinflammatory myokines induces muscle degradation and exacerbates systemic inflammation (12). Nevertheless, further studies are needed to elucidate the mechanism between inflammation and sarcopenia.

The patients with sarcopenia in this study showed a high level of inflammation related markers such as CRP and NLR, suggesting that sarcopenia and inflammation are markers of aggressive tumors (44). Similar results have been reported in head and neck cancers. Cho et al. showed that sarcopenia accompanied by systemic inflammation was significantly associated with poor OS and PFS. In addition, the patients with sarcopenia showed more frequent treatment interruptions due to muscle wasting and their inability to endure the treatment adverse effects (17).

In our study, sarcopenia accompanied by systemic inflammation showed inferior OS and PFS. The poor treatment outcome could be explained by the inability of these patients to tolerate the treatment. Consistent with this notion, sarcopenia did not lower the OS and PFS among the patients who received GP chemotherapy which is the first line chemotherapy.

The adipose tissue composition of patients, VATI and SATI, was not associated with long-term survival in our study. However, there are reports that have demonstrated that high visceral fat is associated with poor survival in cancer patients (45). This discrepancy could be explained by the fact that there are significantly less obese patients in Asia than in western countries. In the other studies, overweight, or obese patients accounted for over half of the total patient population (46, 47). In contrast, only 26.1% of our patients were overweight. Therefore, VATI could affect the prognosis of Asian cancer patients; however, this study does not have the power to determine whether or not it does as the number of patients with high VATI included in this study was insufficient.

There are some limitations in this study. First, the result should be interpreted with caution due to its retrospective nature. For example, though there was an association between sarcopenia, systemic inflammation, and survival, we were unable to define a causal relationship. Second, only Korean patients were included in this study. The skeletal muscle mass varies depending on the disease status and ethnicity of patients. For this reason, diverse cut off values for sarcopenia were used. In this study, we adopted the sarcopenia definition created by the Japan JSH. Yet, the optimal cut off value of sarcopenia for Korean cancer patients is yet to be established. To define the criteria for sarcopenia for the Korean population, especially those with malignancy, further studies are necessary. Also, for subgroup analysis, the number of patients received first line GP chemotherapy were too small to show the statistical power. To complement this limitation, study with larger number of patients is needed. Despite these limitations, this is the first study to demonstrate that sarcopenia accompanied by systemic inflammation is associated with poor prognosis for metastatic BTC.

In conclusion, BTC patients with distant metastasis that had sarcopenia and systemic inflammation at diagnosis were associated with poor OS. Exercise, nutritional support, and pharmacological interventions that block muscle atrophy signals or induce muscle hypertrophy could enhance the survival of cancer patients with sarcopenia and inflammation.

## Data Availability Statement

All datasets generated for this study are included in the article/Supplementary Material.

## Ethics Statement

The studies involving human participants were reviewed and approved by Institutional review board of the Gangnam Severance Hospital. Written informed consent for participation was not required for this study in accordance with the national legislation and the institutional requirements. Written informed consent was not obtained from the individual(s) for the publication of any potentially identifiable images or data included in this article.

## Author Contributions

BL, HJ, and IL: study concept and design, manuscript preparation, data acquisition, and data quality control. BL, YC, HJ, and IL: data analysis and interpretation. YC and JK: manuscript review. All authors: contributed to the article and approved the submitted version.

## Conflict of Interest

The authors declare that the research was conducted in the absence of any commercial or financial relationships that could be construed as a potential conflict of interest.

## Supplementary Material

Supplementary Figure 1 Overall survival among patients who received GP chemotherapy depending on sarcopenia.

Supplementary Figure 2 Progression free survival among patients who received GP chemotherapy depending on sarcopenia.

Supplementary Figure 3 Overall survival among patients who received GP chemotherapy depending on NLR.

Supplementary Figure 4 Progression free survival among patients who received GP chemotherapy depending on NLR.

Supplementary Table 1 Univariate and multivariate analysis of OS using GP chemotherapy.

Supplementary Table 2 Univariate and multivariate analysis of PFS using GP chemotherapy.
