# Supplementary figures and images for: Prognostic Significance of Sarcopenia in Advanced Biliary Tract Cancer Patients
Source: Front Oncol. 2020 Sep 2;10:1581. doi: 10.3389/fonc.2020.01581 (PMC7492547; doi:10.3389/fonc.2020.01581)

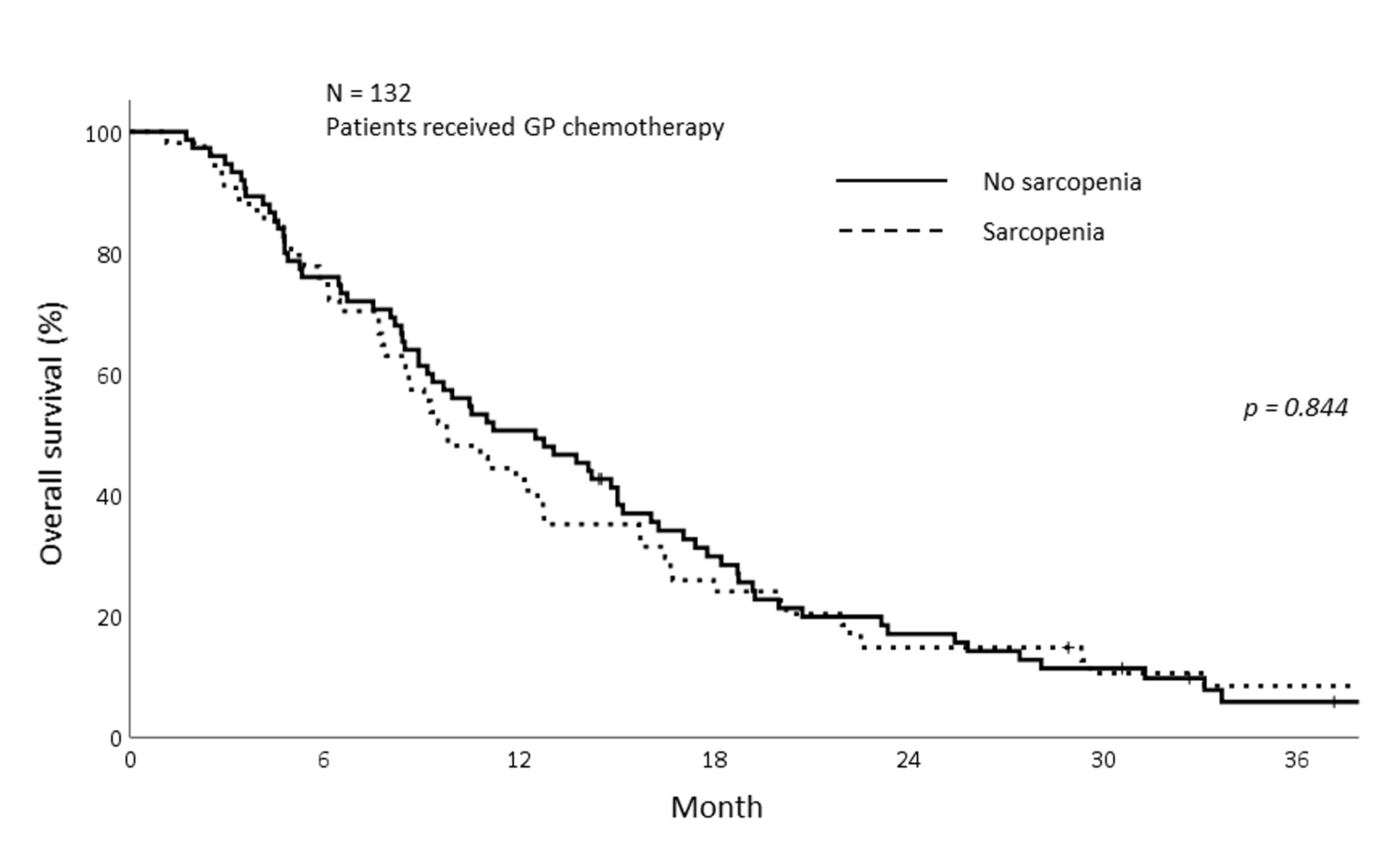

Supplement: Supplementary Figure 1 — Overall survival among patients who received GP chemotherapy depending on sarcopenia. [file Image_1.tif]

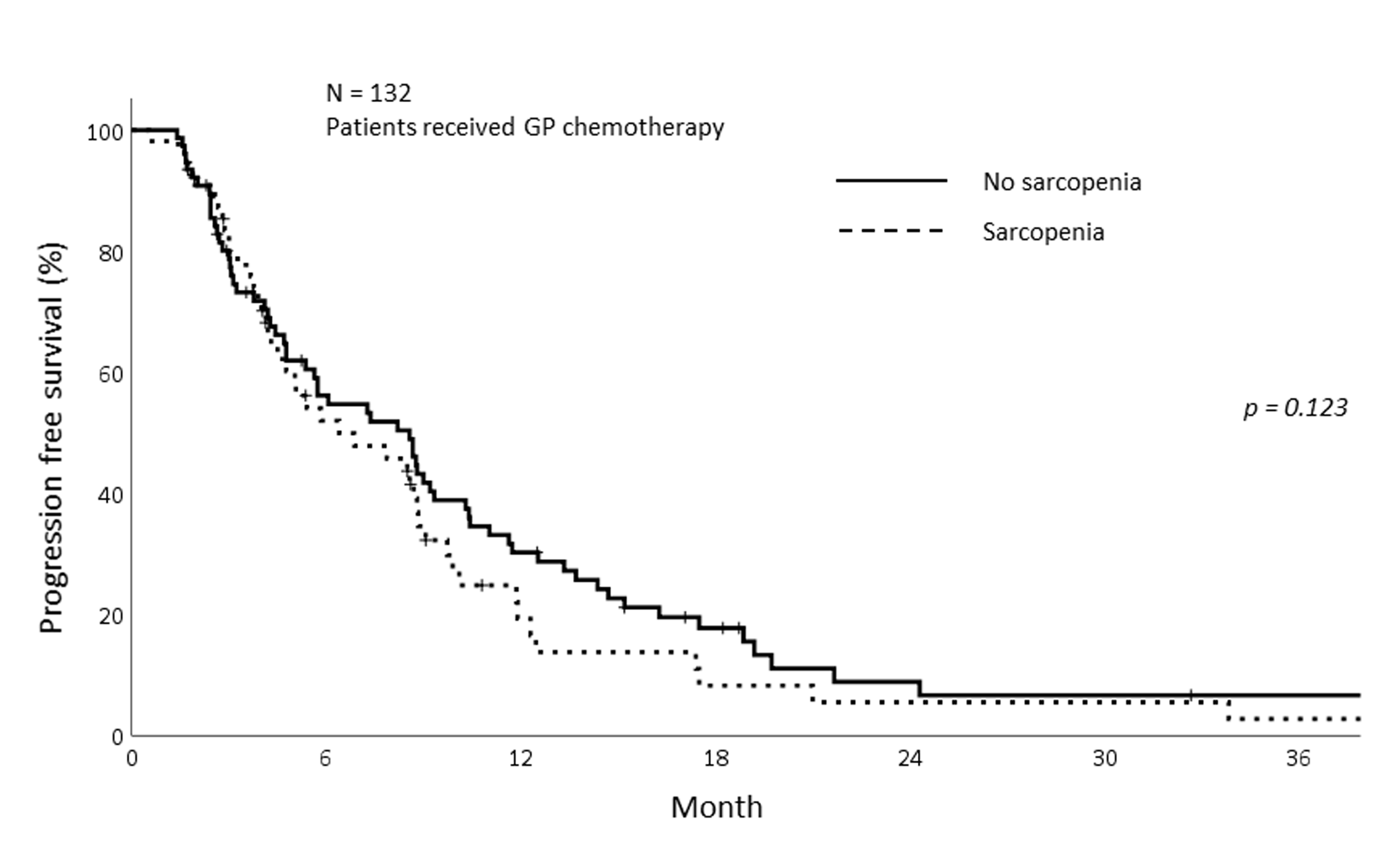

Supplement: Supplementary Figure 2 — Progression free survival among patients who received GP chemotherapy depending on sarcopenia. [file Image_2.tif]

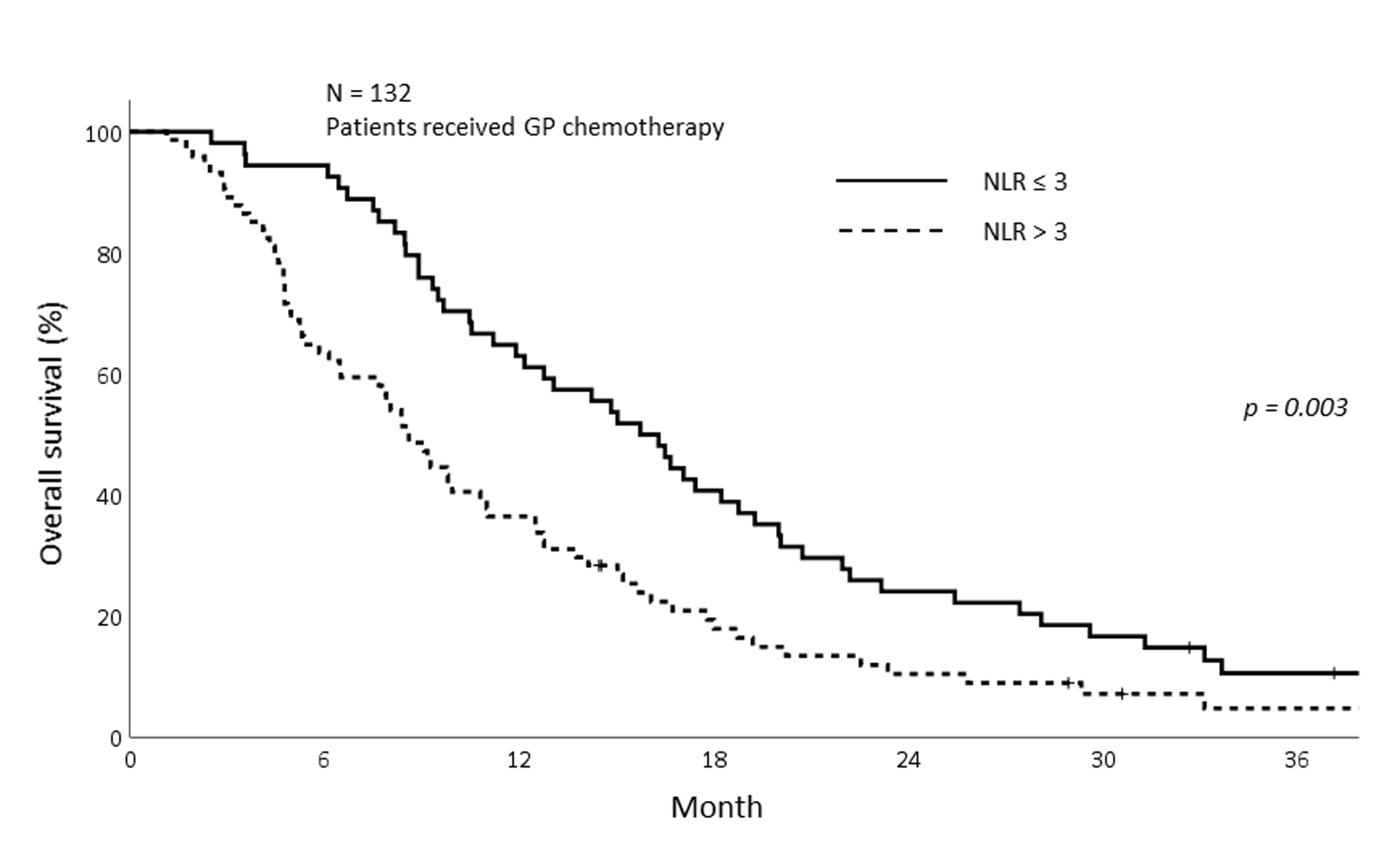

Supplement: Supplementary Figure 3 — Overall survival among patients who received GP chemotherapy depending on NLR. [file Image_3.tif]

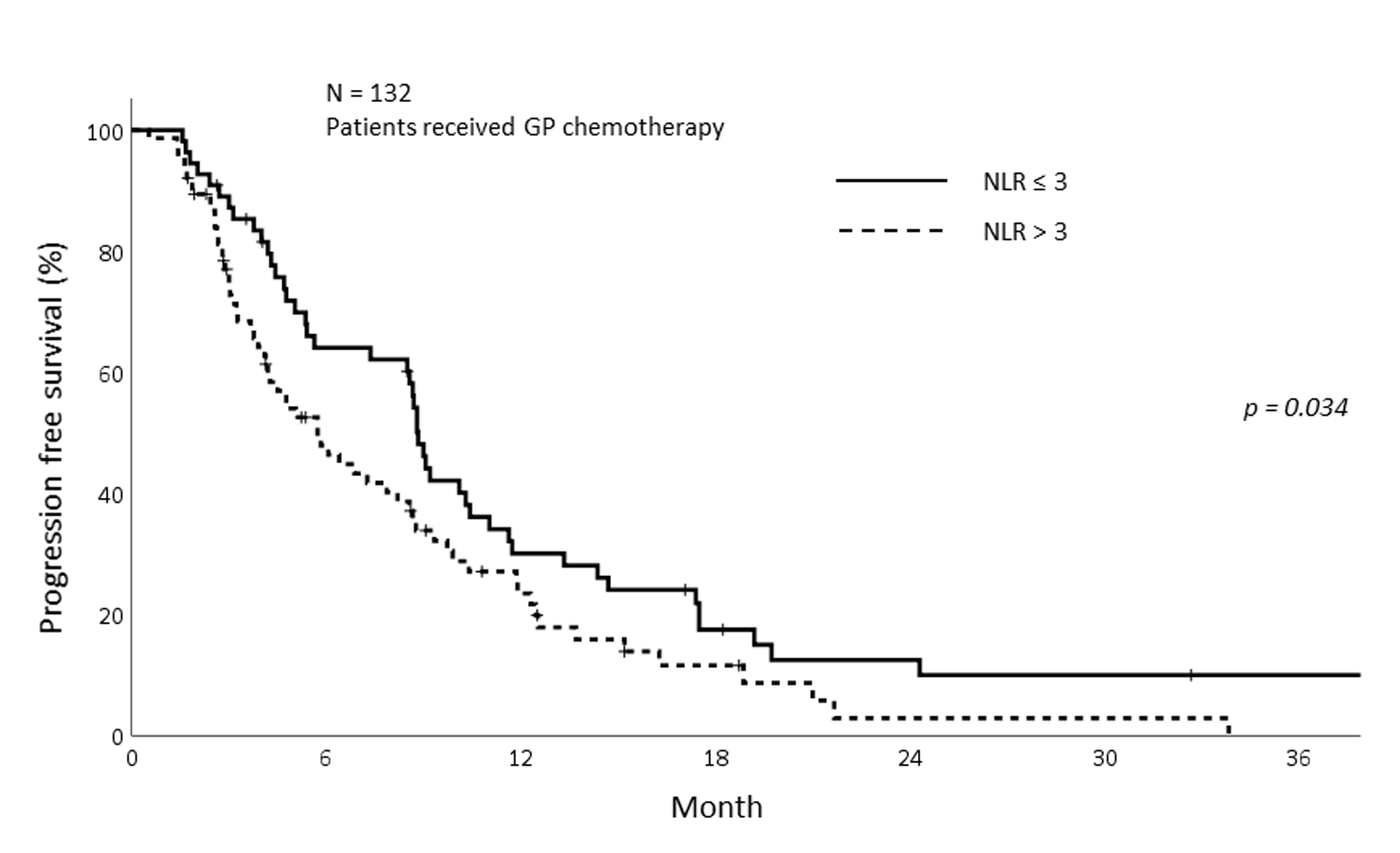

Supplement: Supplementary Figure 4 — Progression free survival among patients who received GP chemotherapy depending on NLR. [file Image_4.tif]
