# Supplementary material for: Prognostic Significance of Sarcopenia in Advanced Biliary Tract Cancer Patients
Source: Front Oncol. 2020 Sep 2;10:1581. doi: 10.3389/fonc.2020.01581 (PMC7492547; doi:10.3389/fonc.2020.01581)
Supplement: Supplementary Table 1 — Univariate and multivariate analysis of OS using GP chemotherapy. [file Table_1.DOCX]

| Supplementary table 1. Univariate and multivariate analysis of OS using GP Chemotherapy | | |  |  |  |  |
| --- | --- | --- | --- | --- | --- | --- |
|  | Univariate analysis | | | Multivariate analysis | | |
| Variables | HR | 95% CI | p-value | HR | 95% CI | p-value |
| Sex (Male vs Female) | 1.70 | 1.18 - 2.46 | 0.005 | 1.63 | 1.11 - 2.39 | 0.013 |
| Age (<67 vs ≥67) | 0.75 | 0.52 - 1.08 | 0.122 |  |  |  |
| Pathology (WD/MD vs PD) | 1.26 | 0.81 - 1.95 | 0.312 |  |  |  |
| ECOG |  |  | <0.001 |  |  | <0.001 |
| ECOG 0 vs ECOG 1 | 2.40 | 1.14 - 5.07 | 0.022 | 2.25 | 1.06 - 4.78 | 0.034 |
| ECOG 0 vs ECOG 2 | 2.57 | 1.21 - 5.46 | 0.014 | 2.18 | 1.02 - 4.68 | 0.044 |
| ECOG 0 vs ECOG 3 | 72.33 | 12.43 - 421.05 | <0.001 | 76.85 | 12.86 - 459.33 | <0.001 |
| ECOG 0 vs ECOG 4 | 2.82 | 0.35 - 22.83 | 0.332 | 2.80 | 0.34 - 23.15 | 0.341 |
| CA 19-9 (per 100) | 1.01 | 1.00 - 1.01 | 0.001 |  |  |  |
| CEA (per 20) | 1.01 | 1.00 - 1.01 | 0.015 | 1.01 | 1.00 - 1.01 | 0.026 |
| CRP (Normal vs Elevated) | 1.93 | 1.30 - 2.88 | 0.001 |  |  |  |
| Albumin (≥ 3.4 vs < 3.4) | 1.72 | 1.06 - 2.79 | 0.029 |  |  |  |
| Protein (≥ 6.9 vs < 6.9) | 1.31 | 0.90 - 1.89 | 0.154 |  |  |  |
| Cholesterol (< 139 vs ≥ 139) | 0.77 | 0.49 - 1.21 | 0.256 |  |  |  |
| BUN (< 23.0 vs ≥ 23.0) | 0.97 | 0.49 - 1.91 | 0.924 |  |  |  |
| Bilirubin (< 1.2 vs ≥ 1.2) | 1.08 | 0.75 - 1.56 | 0.668 |  |  |  |
| NLR (< 3.00 vs ≥3.00) | 1.74 | 1.21 - 2.52 | 0.003 | 1.60 | 1.08 - 2.36 | 0.019 |
| Sarcopenia (Yes vs No) | 0.96 | 0.67 - 1.39 | 0.844 |  |  |  |
| BMI (<25 vs ≥25) | 0.89 | 0.60 - 1.32 | 0.554 |  |  |  |
| VATI (Low vs High) | 1.16 | 0.80 - 1.67 | 0.431 |  |  |  |
| SATI (Low vs High) | 0.95 | 0.66 - 1.37 | 0.788 |  |  |  |
| Abbreviation: HR, Hazard ratio; CI , Confidence interval; CBD, Common bile duct; CCC, Cholangiocarcinoma; GB, Gallbladder; WD, Well differentiated; MD, Moderately differentiated; PD, Poorly differentiated; ECOG, Eastern Cooperative Oncology Group; CA 19-9, Carbohydrate antigen 19-9; CEA, Carcinoembryonic antigen; CRP, C-reactive protein; BUN, Blood urea nitrogen; NLR, Neutrophil lymphocyte ratio; VATI, Visceral adipose tissue index; SATI, Subcutaneous adipose tissue index | | | | | | |

| Supplementary table 2. Univariate and multivariate analysis of PFS using GP Chemotherapy | | |  |  |  |  |
| --- | --- | --- | --- | --- | --- | --- |
|  | Univariate analysis | | | Multivariate analysis | | |
| Variables | HR | 95% CI | p-value | HR | 95% CI | p-value |
| Sex (Male vs Female) | 1.59 | 1.11 - 2.26 | 0.011 |  |  |  |
| Age (<67 vs ≥67) | 0.74 | 0.52 - 1.06 | 0.101 |  |  |  |
| Pathology (WD/MD vs PD) | 1.57 | 1.02 - 2.41 | 0.042 | 1.60 | 1.03 - 2.49 | 0.036 |
| ECOG |  |  | 0.033 |  |  |  |
| ECOG 0 vs ECOG 1 | 2.00 | 1.01 - 3.93 | 0.045 |  |  |  |
| ECOG 0 vs ECOG 2 | 1.76 | 0.88 - 3.49 | 0.108 |  |  |  |
| ECOG 0 vs ECOG 3 | 11.62 | 2.41 - 55.92 | 0.002 |  |  |  |
| ECOG 0 vs ECOG 4 | 1.20 | 0.15 - 9.49 | 0.860 |  |  |  |
| CA 19-9 (per 100) | 1.01 | 1.00 - 1.01 | 0.002 | 1.00 | 1.00 - 1.01 | 0.005 |
| CEA (per 20) | 1.00 | 0.99 - 1.01 | 0.097 |  |  |  |
| CRP (Normal vs Elevated) | 1.69 | 1.15 - 2.46 | 0.007 |  |  |  |
| Albumin (≥ 3.4 vs < 3.4) | 1.47 | 0.92 - 2.33 | 0.104 |  |  |  |
| Protein (≥ 6.9 vs < 6.9) | 1.31 | 0.92 - 1.88 | 0.137 |  |  |  |
| Cholesterol (< 139 vs ≥ 139) | 0.81 | 0.53 - 1.26 | 0.353 |  |  |  |
| BUN (< 23.0 vs ≥ 23.0) | 0.40 | 0.38 - 1.48 | 0.402 |  |  |  |
| Bilirubin (< 1.2 vs ≥ 1.2) | 0.99 | 0.70 - 1.41 | 0.949 |  |  |  |
| NLR (< 3.00 vs ≥3.00) | 1.62 | 1.13 - 2.32 | 0.009 | 1.56 | 1.06 - 2.30 | 0.025 |
| Sarcopenia (Yes vs No) | 0.76 | 0.53 - 1.08 | 0.126 |  |  |  |
| BMI (<25 vs ≥25) | 0.87 | 0.59 - 1.28 | 0.476 |  |  |  |
| VATI (Low vs High) | 1.08 | 0.76 - 1.53 | 0.686 |  |  |  |
| SATI (Low vs High) | 0.86 | 0.60 - 1.22 | 0.397 |  |  |  |
| Abbreviation: HR, Hazard ratio; CI , Confidence interval; CBD, Common bile duct; CCC, Cholangiocarcinoma; GB, Gallbladder; WD, Well differentiated; MD, Moderately differentiated; PD, Poorly differentiated; ECOG, Eastern Cooperative Oncology Group; CA 19-9, Carbohydrate antigen 19-9; CEA, Carcinoembryonic antigen; CRP, C-reactive protein; BUN, Blood urea nitrogen; NLR, Neutrophil lymphocyte ratio; VATI, Visceral adipose tissue index; SATI, Subcutaneous adipose tissue index | | | | | | |
